# Key technology development needs and applicability analysis of renewable energy hybrid technologies in off-grid areas for the Rwanda power sector

**DOI:** 10.1016/j.heliyon.2020.e03300

**Published:** 2020-01-27

**Authors:** Jean De Dieu Niyonteze, Fumin Zou, Godwin Norense Osarumwense Asemota, Samuel Bimenyimana, Gilbert Shyirambere

**Affiliations:** a Fujian Province Key Laboratory of Automotive Electronics and Electric Drive, Fujian University of Technology, Fuzhou, 350118, China; b University of Rwanda, African Centre of Excellence in Energy for Sustainable Development, Kigali, Rwanda; c ShunDe SYSU Institute for Solar Energy, China; d Department of Electrical Engineering, Hebei University of Technology, Tianjin, 300130, China; e Fujian University of Technology, Electrical Engineering, Fuzhou, Fujian, China

**Keywords:** Energy, Electrical engineering, Off-grid rural electrification, Renewable energy, Hybrid systems, HOMER, Rwanda

## Abstract

Until recently, the Rwanda power sector increased rapidly to double the 2010 installed capacity. The energy consumption in Rwanda experienced a steady rise correspondingly with the population and modern socio-economic life. Consequently, Rwanda household access to electricity increased to 53% by September 2019. Not only does 47% of Rwanda's population lack electricity access, there are persistent power failures and the grid is also unstable. Using renewable energy hybrid technologies in off-grid areas might be a solution to this problem. However, the high cost of renewable energy hybrid systems has led to its slow adoption in many developing countries. Hence, it is important to find the most appropriate hybrid combinations that reduce energy cost and access electricity generation that maximizes the available renewable energy resources. This paper examines some new technology development needs related to the power sector in Rwanda. Secondly, four different 100% renewable energy hybrid systems were designed and simulated to support rural and remote areas considering an average load demand of 158.1 kWh/day with a peak load of 18 kW. The hybrid systems simulation and optimization were obtained using HOMER (hybrid optimization model for electric renewables) software. The input data were obtained from National Aeronautics and Space Administration (NASA) for solar and wind resources, and hydro resources were from real-time field data for selected study site. The simulation results indicate hydro/solar/battery hybrid is the most cost-effective and environmentally viable alternative for off-grid rural electrification because of low net present cost (NPC) and least greenhouse gas emissions. The proposed hybrid combination could apply to other rural areas in the region and elsewhere in the world especially where climate conditions are similar.

## 1 Introduction

Rwanda is an East African country enclosed by the Democratic Republic of Congo (DRC) in the west, Tanzania in the east, Uganda in the north, and Burundi in the south. Its undulating hills and lush landscape enable it to accommodate an estimated 12.2 million population (2017) in an area of 26,338 square kilometres (Km^2^). Also, it is one of the most densely populated countries in Africa [1, 2]. In the last two decades, Rwanda has made significant progress in her economic prosperity and human development indices. However, providing reliable and cost-effective energy remains one of the major challenges it will face in the 21st century [3, 4].

The energy sector input is a stimulant and an incentive that enables the productive sector to significantly contribute to economic growth and development. This makes the government ensure and continuously strive to power Rwanda cities and villages with reliable, efficient and affordable power to improve living standards for all its population. Further, modern energy is a key success factor in enhancing development and prosperity through the provision of lighting, heating, transports, communications, and mechanical power services. The success of rural electrification programmes has been limited because over 1 billion people worldwide cannot access electricity. Specifically, over 600 million people in Sub-Saharan Africa cannot access electricity [5].

Although access to modern energy has been indispensable to improved education, higher quality of health and higher standards of living, lack of electricity access has led to more deaths and illnesses per year as a result of air pollution in households, which use traditional stoves [6]. Even though grid extension is preferred for rural electrification in Rwanda, changes to the operation modes and exploitation of electricity are necessary. Renewable energy (RE) experts agree that off-grid energy resources can significantly address the country's power access. Therefore, renewable systems such as solar, hydropower and wind turbines were recommended by researchers after major experiments [7, 8, 9, 10]. Moreover, RE is not the only source of energy but it can generate clean energy solutions which reduce the health and environmental impacts associated with greenhouse gas emissions.

The purpose of this paper is twofold: (a) to recommend a set of power sector key technologies development needs in the Rwanda power sector. There can be no doubt that implementing some new technologies is one of the biggest solutions to power sector challenges facing the country today, (b) to examine RE hybrid combinations suitable for different off-grid areas based on their natural resources. Thereafter, the data will be analysed by inputting different sets of data in HOMER (hybrid optimization model for electric renewables) software. Also, the best option based on the cost of electricity generation compared to the grid extension-related costs will be selected in the study.

The paper has been organized into: Introduction, Literature review of the energy sector in Rwanda and its technological needs, Methodology, Simulation, optimization results and discussion and Conclusion.

## 2 Literature review of energy sector in Rwanda and its technological needs

The purpose of this literature review is three-fold: First, it shows the summary of the power sector in Rwanda. It provides evidence for this work by justifying the energy shortage the country is currently experiencing. Secondly, it indicates Rwanda power sector has technological development needs. The reason for this is that the integration of clean energy technologies into the grid worldwide is changing fast. In developing countries like Rwanda with incessant power outages, sustainable energy development and clean energy development implementation, need careful planning especially because of the financial implications. Lastly, experience in developing countries shows how HOMER software was used to study different optimal hybrid systems that combine engineering and economic solutions into optimal models that enable users determine least-cost options.

### 2.1 Energy resources and consumption in Rwanda

Rwanda has significant hydropower generation potential because of its topography. It is ideal for medium-to high-head pico and micro-hydro run-of-river projects [11]. Besides hydropower, methane gas, peat, geothermal, solar, biomass and wind, there are other viable primary energy resources in Rwanda. However, assessment of wind power resources is at its infancy. Therefore, prospecting will continue so that wind power can contribute to the generation [12].

**Figure 1:**
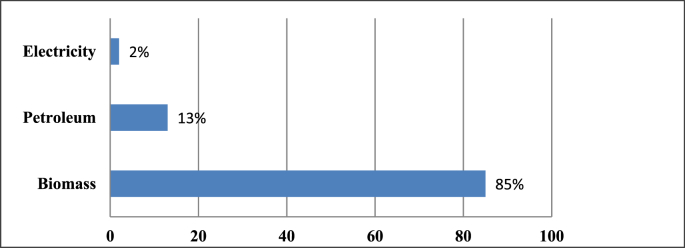
Energy consumption in Rwanda in percentages (%) (2016).

**Figure 2:**
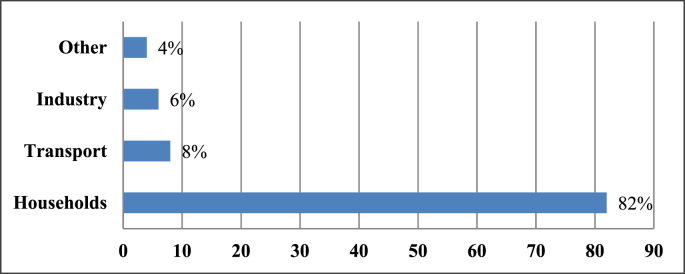
Energy consumption per user category in Rwanda in percentage (%) (2016).

The energy consumption by sector in Rwanda is presented in Figure 1. Of the total energy consumption, biomass, petroleum, and electricity account for 85.0%, 13.0%, and 2.0%, respectively [12]. Moreover, Figure 2 shows the energy consumption per user category which is led by households, transport, industries and others at 82.0%, 8.0%, 6.0% and 4.0%, respectively [12].

### 2.2 Rwanda energy generation capacity

**Figure 3:**
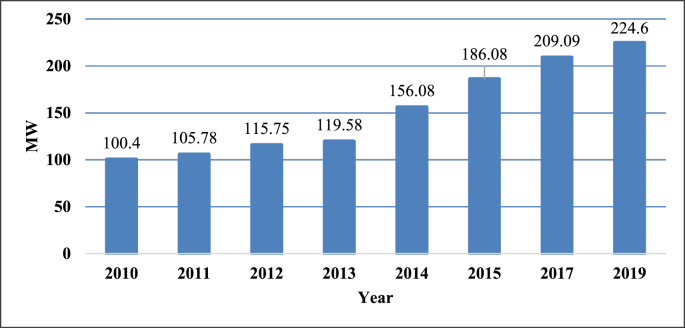
Evaluation of the installed generation capacity on the national grid in MW.

Rwanda is rich in natural energy resources like hydro, geothermal, solar, and methane gas. Throughout the site visits to the National Electricity Control Centre, the installed power generation capacity was 224.6 megawatts (MW) as shown in Figure 3. Only 11.0% of the available capacity is imported while the remainder is generated locally. According to Rwanda Energy Group (REG), the power generation capacity increased to 224.6 MW in October 2019 [13,14]. The national electrification rate was 53 per cent and generation technology mix was: 39.0% hydrological resources, 25.0% methane gas, 19.0% thermal sources, 4.0% peat, 2.0% solar and 11.0% imports from neighbouring countries to strengthen energy capacity [13, 14].

### 2.3 Key technology development needs in the Rwanda power sector

The special economic zones were established by the government of Rwanda as key policy instruments to overcome constraints of scale and competitiveness in the provision of strong enabling environments [15]. Access to reliable and affordable electricity is critical for economic growth and continued prosperity for the people. However, the electricity sector faces challenges and therefore, sustainable RE integration is needed for flourishing business climate and overall welfare. In this paper, we also focus on new sustainable energy technologies so that energy efficiency and energy generation can be both improved.

#### 2.3.1 Advanced metering infrastructure

**Figure 4:**
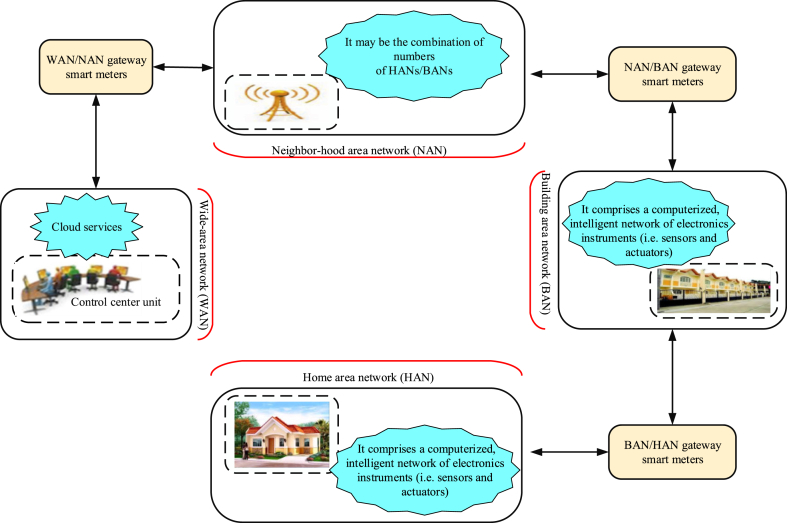
Hierarchical architecture of advanced metering infrastructure (AMI).

Advanced metering infrastructure (AMI) system comprises integrated technologies and applications that include: smart meters, wide-area communications infrastructure, home (local) area networks (HANs), meter data management systems (MDMS), and operational gateways. Although the country continues to invest in different energy technologies, AMI can be used to improve energy efficiency service delivery to produce favourable environmental impact [16, 17]. Figure 4 graphically describes how from communicational and security perspective, what components constitute an AMI (see Figure 4). Hence, it clearly shows that each application or layer is important in determining suitable technology for smart grid deployment.

#### 2.3.2 Advanced distribution automation

Advanced distribution automation (ADA) system manages and controls distribution systems for completely controllable and automated distribution. ADA provides complete infrastructure integration and distributed resources technology that increase reliability and more efficient operation of the entire system [17, 18, 19, 20].

The management of electrical power in Rwanda is accomplished by calling on different power plants and substations, to balance the consumption of various feeders using the supervisory control and data acquisition (SCADA) system. Consequently, ADA positively influences grid reliability, management, growing demand, efficiency, service quality expectations, security, sustainable environment and overall sustainability of the energy sector.

#### 2.3.3 Intelligent universal transformer

Intelligent universal transformer (IUT) was proposed by the Electrical Power Research Institute (EPRI) to replace conventional distribution transformer with the latest power electronic systems [21]. The individually distinct functional units enable users to switch to different voltage or power levels.

**Figure 5:**
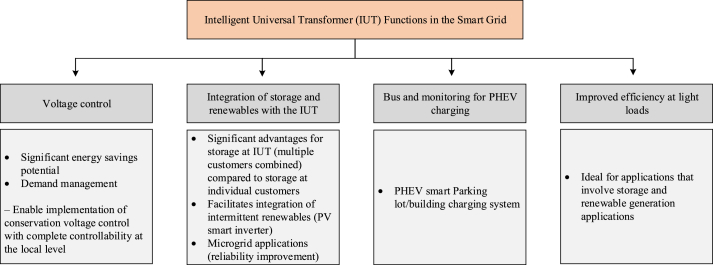
Functions of IUT in the smart grid.

The major attributes of IUT include [22, 23]: Instantaneous voltage regulation under dynamical loads and transients, maintaining unity input power factor under reactive loads, maintaining clean input under harmonically distorted or nonlinear loads, protection against unbalanced loads, protection against overloads and output short-circuits, voltage sag compensation, outage compensation, and capacitor switching protection. The IUT is a promising new technology that can impact a wide range of applications especially based on RE systems. The functions of IUT in the smart grid are summarized in Figure 5.

#### 2.3.4 Distributed energy resources-smart grid integration

Some of the distributed energy resources (DER) include local renewable energies, advanced inverters, and energy storage. Smart grid possibilities to the community are new grid operations that accomplish more sustainable, secure, and cost-effective energy systems for long-term power backup in prioritized loads [24, 25, 26].

#### 2.3.5 Demand response

Demand response (DR) measures rate of electrical energy supply to consumers that motivate electricity end-use changes by customers in response to electricity price elasticity over time. It could be incentive payments to encourage lower electricity use at times of higher market prices or when grid reliability is threatened [27, 28].

DR and energy efficiency affect customer energy end-use. The most important benefit of DR is the strong impacts on energy policy pillars associated with economic, environmental, and reliability objectives [29]. Additionally, DR decreases consumption during peak times and leads to peak clipping. Load shifting is associated with usage reduction during peak that is offset by usage in off-peak hours [30, 31].

#### 2.3.6 Open automated demand response standard

Recent advancements in science and technology made it possible to integrate AMI energy systems and services for two-way interaction. Thus, AMI is used in smart grids for load management and DR. However, smart grid devices produce large data, which need sizeable storage capacity and processing power for analyses. The open automated demand response (OpenADR) standard using AMI support in cloud computing environments assess crucial parameters that send and receive DR signals [32, 33].

OpenADR was accepted by the United States (U.S.) government research laboratory for research and standards development efforts. It is a completely automated DR using open standard, platform-independent and transparent end-to-end technologies or software systems [34, 35, 36].

#### 2.3.7 A large scale integration of electrical vehicle

Electric vehicle (EV) and plug-in hybrid electric vehicle (PHEV) are the future of transportation and indispensable building blocks of the energy transition. EV and PHEV batteries are charged or discharged using simple plug-in connectors compatible with local electricity distribution grid [37]. EV integration can address the energy crisis, improve air quality and decrease carbon footprint [38].

Since 2017 the government of Rwanda introduced plans of assembling EV as part of the environmentally friendly model for vehicles. This is so because different studies have revealed that vehicles emissions have a major contribution to air pollution in Rwanda. The choice of EV could prove good to Rwanda because fuel is expensive. It is imported from the Middle East through Tanzania, travelling nearly 3000 km, before reaching Kigali [39].

#### 2.3.8 A large scale integration of wind energy

The process of developing wind energy as a significant source of world's energy should be the priority for important developments and implementation of the 21^st^ century [40]. There is a great interest in harnessing wind as a new source of power in Rwanda. The development of wind energy needs detailed and reliable information on wind regimes and potential exploitation sites which have not yet been mapped out in Rwanda. However, energy consumption in Rwanda has shown a continuous rise. The energy sector should explore new energy resources including the possibility of wind energy as much as possible.

Previous research shows that wind integration in Rwanda needs policymakers and extensive investigations based on a more detailed and systematic analysis of wind speed patterns. Moreover, the available annual average wind speeds in some areas of the country including Kigali City can be used to generate electricity in windy seasons [7].

### 2.4 Reference examples of hybrid energy systems analysis using HOMER

Installing huge numbers of RE hybrid systems would have a major impact on climate change which is an excellent approach that needs to be sustainable to provide energy access for all citizens. The evidence of other researches is used to show how the exploitation potentials of renewable-hybrid energy options for off-grid areas in different areas have progressed. Thus, it offers a better regional understanding of renewable energy exploitation, uses and technologies for harvesting the resource. This is so because its successful implementation in Rwanda can easily be exported to permeate the East African Region (Community), as first beneficiaries of the research and other countries without access to electricity for all her people.

Apart from gaining access to RE and HBT, experience in developing countries also shows how researchers have studied different optimal hybrid combinations that suit off-grid villages in several applications using HOMER software. Thus, researchers all over the world have expended great efforts to develop renewable energy sources like wind and solar power in different case studies using this software. For example, Singh and Baredar presented a study of India [41]; Halabi and Mekhilef discussed the case of Malaysia [42], and also, Shezan and other co-authors, provided different case studies using optimal hybrid system combinations [43, 44]. Doubtless, off-grid communities lack regular access to electricity. Hence, reliable independent systems powered by wind, water, solar, and other resources could hold the key to a solution. Table 1 summarizes the technology, case studies, technology applications, and objectives of these studies.

**Table 1:** Examples of hybrid energy systems analysis using HOMER software.

Authors& references	Technology application	Case study	Objectives of the study
Singh A. and Baredar, P. [41]	Solar, fuel cell, and biomass	India	Computation, simulation & optimization of a hybrid system using biomass gasifier generator set, solar and fuel cell with battery
Halabi, L. M. and Mekhilef, S. [42]	Hybrid PV/diesel/battery	Malaysia	Verification of site data sets, simulation of different operational scenarios, and a comparison with optimum design
Shezan, S. K. A. and Al-Mamoon [43]	Combination of renewable and non-renewable resources	Indonesia	Performance investigation of an advanced hybrid energy system in rural and remote areas
Shezan, S. A. and Saidur, R. [44]	Wind, diesel generator and batteries	Malaysia	Feasibility analysis of a hybrid off-grid wind-diesel generator-battery system for eco-tourism in remote areas

## 3 Methodology

### 3.1 Introduction

This paper uses literature review, data collection from government energies institutions and HOMER software to analyse different renewable hybrid systems for integration into Rwanda off-grid areas. Moreover, we used the updated input data for software simulation as the study strategy for this hybrid technology (HBT) assessment.

HOMER was used to design different models that indicate how various natural RE sources are combined for clean energy generation. Data were inputs from the off-grid sector and analysed using HOMER.

To ensure validity and reliability of the study, we collaborated with several power plants owners and operators in the initial and final stages of the research. According to these experts, policy on rural electrification implementation strategy lacks pertinent guidelines, created risks, caused unfavourable market distortions, and made feasibility studies of new power plants to be necessary.

**Figure 6:**
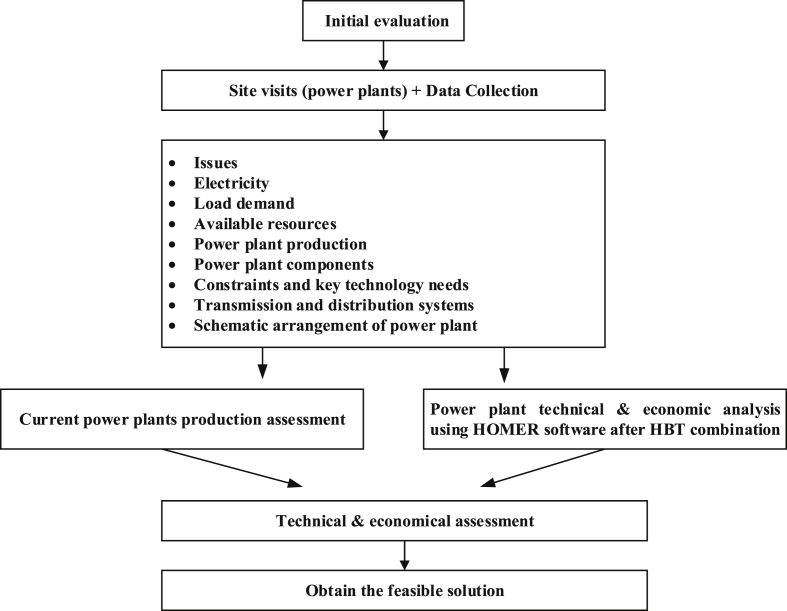
Framework for analysis of the study.

The basic framework of this paper is shown in Figure 6. Based on the site visits and data collection conducted by our team, all the research needs were taken into consideration. These include electricity load demand, available resources, power plant production, power plant components, constraints and key technology needs, schematic arrangement of power plants, transmission and distribution. Consequently, the three power plants visited were assessed for technical and economic feasibilities using HOMER software after HBT combination was compared. The objective of these technical and economic assessments was to obtain a feasible HBT combination that suits off-grid areas of the Rwandan power sector at minimum cost and maximum availability.

### 3.2 HOMER software

**Figure 7:**
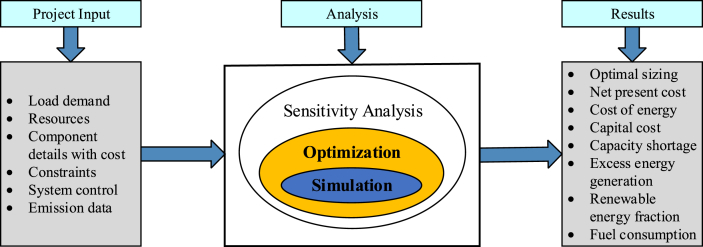
Schematic representation of HOMER software.

HOMER software is the hybrid power system optimization software developed by the U.S. National Renewable Energy Laboratory (NREL). It is used to design micro-power systems that facilitate comparison of power generation technologies across broad applications [45]. Figure 7 shows a detailed schematic representation of HOMER. It performs three principal tasks: simulation, optimization, and sensitivity analysis. It can effectively perform energy balance calculations for each system configuration using its three core capabilities. HOMER uses inputs like load profile, site-specific resources and system components.

In the simulation model, HOMER attempts to simulate a viable system for all possible combinations of equipment considered. The step that follows every simulation is optimization. All simulated systems are sorted and filtered according to defined criteria to obtain the best possible fits. However, sensitivity analysis is optional but helps HOMER users model the resources variables beyond their control like wind speeds and fuel costs. It enables researchers observe how the optimal system changes with such variations [45, 46]. The optimization oval encloses the simulation oval indicating that a single optimization consists of multiple simulations. Similarly, the sensitivity analysis oval encloses the optimization oval because a single sensitivity analysis consists of multiple optimizations, which is shown in Figure 7. HOMER also displays simulation results in a wide variety of tables and graphs which helps to compare configurations and evaluate them on their economic and engineering merits such as system sizing, performance details, financials and various reports.

### 3.3 Data collection

In this study, the designed questionnaire was used to collect data from 101 households in 4 provinces of Rwanda except Kigali City. This survey was conducted by asking local people several questions. The data were analysed using statistical package for the social sciences (SPSS) version 23.0. As a result, the electricity demand of each house and the total energy consumption of people who live in off-grid areas were estimated. The purpose of survey was to estimate the demand of consumers which is the basic requirement for designing a power station. Additionally, during the research, three mini-grids were visited and their data were collected.

To analyse solar and wind power integration in off-grid areas, the wind speed data and solar irradiations during a year are needed. These data were obtained from Rwanda Meteorology Agency, a public institution that provides accurate, timely weather and climate information and products for the general welfare of the people.

Additionally, the other wind speed and solar irradiations data were obtained from The National Aeronautics and Space Administration (NASA)'s surface solar energy data set which provides monthly average solar radiation data for everywhere on earth [46]. The provided data helped us to compare and to make sure the wind and solar systems integration into the Rwanda power system for this research are credible.

Moreover, we spent 2 months at the Rwanda National Electricity Control Centre in 2014 where we were trained on the control and generation of electricity in the whole country. Therefore, the data of Rwanda power sector were obtained. In 2017, we again visited the Rwanda National Electricity Control Centre and other installations of REG, to obtain data and information for the study. This makes us to be conversant with the current status of Rwanda power sector and to understand the policies and plans of the Rwanda government in the power sector. However, during the first drafting of this paper these data that were taken before were not updated, that was why we went back to the Rwanda National Electricity Control Centre to collect newly updated data.

### 3.4 Selected sites

The rural electrification strategy was approved by the government of Rwanda by the end of 2016 where the partnership of the government, private sector and development partners have contributed immensely to increase rural electrification, and set lofty targets for the future. As a consequence, pico/mini-hydropower, and solar-powered mini-grids were successfully developed in Rwanda [47].

In this study, we visited 3 mini-grids which sought to provide solutions to off-grid rural electrification since 2016. These sites were chosen for their technology, RE resources and social impacts on the communities in off-grid areas. The descriptions of these three different sites are below.

#### 3.4.1 Mukungu pico-hydropower plant

Mukungu pico-hydropower plant is located in Karongi district, Western province of Rwanda; with Global Positioning System (GPS) coordinates: S 02° 13 931′ and E 29°24.590’. The site construction objective is to provide energy that promotes economic development for up to 400 + households located in the rural area (off-grid zone). Table 2, shows the hydro resources flow rate data which are assessed 2 to 3 times per month so that the sum and average for every month can be calculated. These data were obtained from the owners of this power plant in 2016 (EAPICO Ltd).

**Table 2:** Monthly average flow rate at Mukungu River [EAPICO LTD [47]].

Month 2016	Jan	Feb	Mar	Apr	May	Jun	Jul	Aug	Sept	Oct	Nov	Dec
Streamflow (L/s)	280	334	321	394	327	250	197	340	378	376	372	392

The first part of this plant comprises intake and weir, channel, forebay, penstock and powerhouse, which contains the electromechanical equipment such as cross-flow turbine, alternator and its auxiliaries.

Secondly, there are single and three-phase low voltage (LV) power distribution lines. These lines were constructed using the power line standards established by REG. The 2.5 kilometres (Km) mainline is served by 3-phase. The 1-phase line supplies many customers except for big end-users that need 3-phase power. Finally, all customers served by this plant use the REG wireless smart meters to access their energy services.

The total budget of this project was Euros 279,665 and the expected power generation capacity is 14.7 kilowatt (KW) (an increase from the original 7.0 kW) [48]. However, data collected from the site after completion of the project shows plant capacity to be 21.0kW mechanical, 16.9kW electrical and (United States Dollar) USD 112,960.7 plant cost (without the yearly cost of maintenance of the components (O & M cost)).

Hydropower brings electricity to people in different urban and rural areas. Mukungu pico-hydropower plant provides electricity access for over 400 households, 1 health centre, 1 primary school, 1 secondary school, sector and cell administrative offices, a business centre and a church parish.

#### 3.4.2 MeshPower Gitaraga solar mini-grid

MeshPower is a British energy firm which design and implement solar-powered mini-grids and smart metering systems. They provide affordable and reliable electricity to communities without access to energy in Rwanda.

In 2018, MeshPower completed the installation of the first alternating current/direct current (AC/DC) hybrid mini-grid system at Gitaraga village in Bugesera district, Rwanda (GPS coordinates: 2°04′34.3″S 30°07′12.6″E). The construction objective of this site is to provide productive uses for electricity that engages artisanal customers like welders, tailors, hairdressers and promote economic development at the grassroots.

The site consists of 3 main parts as described below:

(1) Two powerhouses-one is for DC base station which helps to produce DC energy, remotely monitor the two powerhouses and provide free wireless for local customers and the second powerhouse generates AC energy for big end-users.
(2) Overhead single-phase low voltage power distribution line. This line was constructed using the power line standards established by REG
(3) Customer connection-this part supplies power to customers and consists of miniature circuit breaker (MCB) load limiter fixed on the distribution pole and the energy meter serves and monitors the energy used by a customer. In summary, the customer's house supplied by cable serves DC energy for lighting and low power appliances while the AC cables serve big end-users.

Also, Xpower is a U.S. company that focuses on developing technologies and partners to MeshPower. The installation cost of Gitaraga mini-grid is USD 25,000 [49]. The mini-grid provides 10.0-kilowatt peak (kWp) AC power to over 20 small businesses and DC power to over 100 households in the village.

#### 3.4.3 Cyimbiri micro-hydro power plant

ENERGICOTEL was awarded a contract by the government of Rwanda and Energy Utility Corporation Limited (EUCL) to rehabilitate, finance, operate and maintain 5 mini-hydropower plants including Cyimbiri mini-hydropower plant. This contract was to generate and supply power to the national grid as per the power purchase agreements (PPAs) signed in August 2015 (GPS coordinates: 1°49′39.6″S 29°17′54.3″E) [50].

The plant structure is divided into 3 main parts:

(1) Civil works structure including intake/weir, channel and penstock
(2) Powerhouse contains the electromechanical equipment, described below:

The turbine is a cross-flow rated 318 kW mechanical power. It was manufactured by Ossberger of Germany. The 3-phase synchronous alternator connected to turbine has the following specifications: 330 kVA, 50 hertz (Hz), 90% efficiency, 110% in 2 h overload, 400–690 volts (V) variable voltage service, vertical axis; 48 V direct current (VDC) stored by the batteries or backup storage of auxiliary emergency supply.

The controller system board connected to the National Electricity Control Centre uses SCADA.

(3) Switchyard and grid

The alternator (330 kVA) is connected to the step-up transformer 0.4/30 kilovolts (kV) medium voltage (MV) line, which is connected to the national grid.

For hydro resource, below is the hydropower monthly average stream flow data: (data obtained and surveyed on Cyimbiri and Nkora rivers from August 2006 to July 2007).

**Figure 8:**
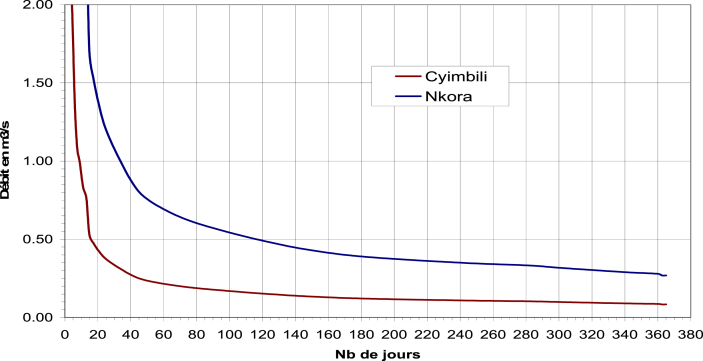
Classification of rivers water-flow rate of Cyimbiri and Nkora rivers. The figure was obtained from Cyimbiri micro-hydro power plant during the visit and data collection.

Figure 8 shows the classification of rivers water-flow obtained as shown below:

Y-axis: Débit (French language): is flow in cubic metres per second (m^3^/s).

X-axis: Nb de jours (French language): is number of days.

**Figure 9:**
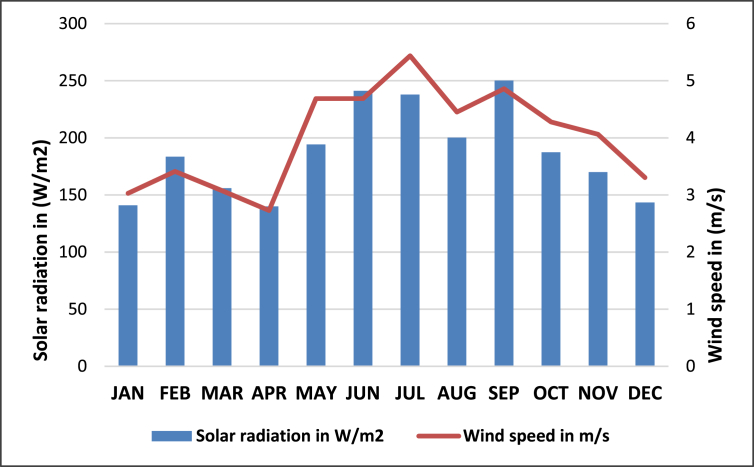
Alternative renewable energy profiles at Rutsiro district (Solar and wind energy resources).

Cyimbiri micro-hydropower plant has contributed to the socio-economic development of the neighbouring communities through job creation and availability of sustainable power supply in the region. The installed capacity of Cyimbiri micro-hydro power plant is 300 kW and the plant cost is USD 1,289,422 [50]. Furthermore, about 46.4 % of the total installed capacity in Rwanda is from hydropower plants consisting of over 40 power plants [14]. For this reason, other non-RE resources should be prioritized in favour of alternative energy sources. In this study, we visited Rwanda Meteorology Agency to check several RE resources data for power plants located in Rutsiro district of Rwanda. Figure 9 below shows several alternative energy resources at Rutsiro district (Solar and wind energy resources).

### 3.5 Off-grid village energy for household basic needs

Energy consumption in domestic households is determined by type and number of electrical appliances in the property and appliances time of use by occupants of the building [51]. In this study, the estimated load was assessed based on a survey conducted in the different villages of the country. A set of questionnaires was designed and validated for reliability by expert judges for data collection from respondents based on their energy consumption patterns. In this survey, 101 houses’ representatives filled out questionnaires concerning their home electrical appliances and monthly energy usages. Statistical analysis of all entered data in SPSS version 23.0 was used to obtain the results in Table 3. Table 3 shows the details of daily energy consumption of 101 households and their respective power rating.

**Table 3:** Estimated daily electricity demand for 101 households in the village.

No.	Equipment in 101 households	No. in use	Power consumption (W)	Total power consumption (W)	Hours of use/Day (Hr)	Watt-hours/day
1.	Lamps	744	10	7440	5	37200
2.	Cell phones	280	10	2800	8	22400
3.	Ceiling fan	0	75	0	0	0
4.	Radio	94	20	1880	5	9400
5.	television	41	120	4920	5	24600
6.	Computer	3	100	300	5	1500
7.	Refrigerator	4	500	2000	24	48000
8.	Iron	15	1000	15000	1	15000
**Daily total energy consumption in 101 households**					**158100Watt-hours/day =158.1kWh/day**	

Abbreviations: W: Watt, kWh: kilowatt-hour, Hr: hours.

In Rwanda, based on extensive energy consumption survey, it shows that remote rural village demand for electricity is not high compared to urban areas. Domestic electricity demand is used in appliances like radios, lamps, cell phones, ceiling fans, electric irons, refrigerators, and computers.

Moreover, the statistically analysed data from questionnaires showed that 43.6% of all households incurred FRW 500 and 21.0% incurred FRW 1000, monthly electricity bills, respectively (Rwanda Francs (FRW)). Since the last electricity tariff revision of 2018, the cost of electricity for residential, non-residual and industrial users depended on usage levels. For the residential category, current electricity tariffs have classified consumers into: 0-15 kWh/month, 15-50 kWh/month, and the bigger consumers over 50 kWh/month, to pay FRW 89 per kWh, FRW 182 per kWh, and RWF 210 per kWh, respectively [3, 52].

Additionally, the percentage of households having home appliances like television sets, radios, electric irons, and refrigerators were 39.6%, 91.1%, 14.9%, and 4.0% respectively. However, modern appliances such as washing machines and ceiling fans were not found in this survey.

### 3.6 RE resources proposed for the HOMER software in Rwanda

An energy resource applies to assets coming from outside the system used to generate electric or thermal power. *Renewable Energy* is any illimited natural energy resource, which can be used continuously. Nevertheless, only three renewable energy sources (biomass, geothermal, and solar) can be used to provide sufficient heat energy for power generation in Rwanda. Arguably, only solar energy promises the greatest global potential because geothermal sources are localised to a few areas and the supply of biomass is not ubiquitous [53].

In HOMER, the resources library contains saved resource definitions which enable us to specify new resources. In the initial HOMER program used for this simulation, only fuels were implemented in the resource library [45]. Rwanda's RE potential sources include wind, solar, hydropower, and geothermal energy [54]. However, wind power in *Rwanda* has not been fully harnessed but there are only two operating small wind power-generating turbines in Rwanda. A wind turbine for pumping water is installed at Gabiro district in Northern Province. Another one was installed at Mont Karisimbi for running frequency modulation (FM) transceiver antenna for national radio and television [55, 56].

### 3.7 Selected hybrid system schematics and components

#### 3.7.1 Modelling approach and selected HBT

Rwanda has a lot of untapped RE resources sites. The resources for electricity generation include hydro, geothermal, methane, peat, solar, wind and waste energy. In 2007, the Outlook report of Rwanda Environmental Management Agency (REMA) showed that a lot of untapped resources for power generation amounted to about 1,200 MW [57, 58]. Unfortunately, most of these resources remain untapped while the total installed capacity for generating electricity in Rwanda is only 224.6 MW by 2019 [14].

HOMER is a very useful software system for simulation, which gives more detailed information than other statistical models. It can perform optimization and sensitivity analysis with limited data [59]. It suggests the best-optimized model design using given inputs based on Net Present Cost (NPC) which results in the most economical output. It simulates system operations by producing energy balance calculations for each of the 8,760 h per annum. Consequently, it determines feasible configurations and estimates the cost of installing and operating the system over the project lifetime [60].

HOMER determines the total annualized costs for each component, using any miscellaneous costs and penalties for pollutant emissions of the system. It uses this value to determine the total net present cost and the Levelized cost of energy (LCOE or COE) [45]. HOMER evaluates the NPC from [45]:

(1) $$C_{NPC}=\frac{C_{ann,tot}}{CRF\left(i,R_{proj}\right)}$$

where ${\text{C}}_{\text{ann},\text{tot}}$ is total annualized cost, i is annual real interest rate (discount rate), ${\text{R}}_{\text{proj}}$ is project lifetime, and CRF(.) is capital recovery factor. CRF is given by [45]:

(2) $$CRF\left(i,N\right)=\frac{i{\left(1+i\right)}^{N}}{{\left(1+i\right)}^{N}-1}$$

where i is annual real interest rate and N is number of years. Also, HOMER uses Eq. (3) to evaluate the Levelized cost of energy [45]:

(3) $$COE=\frac{C_{ann,tot}}{E_{prim}+E_{def}+E_{grid,\;\;sales}}$$

where ${\text{C}}_{\text{ann},\text{tot}}$ is total annualized cost, ${\text{E}}_{\text{prim}}$ and ${\text{E}}_{\text{def}}$ are annual total amounts of primary and deferred load, respectively and ${\text{E}}_{\text{grid},\text{ sales}}$ is annual energy grid sales.

**Figure 10:**
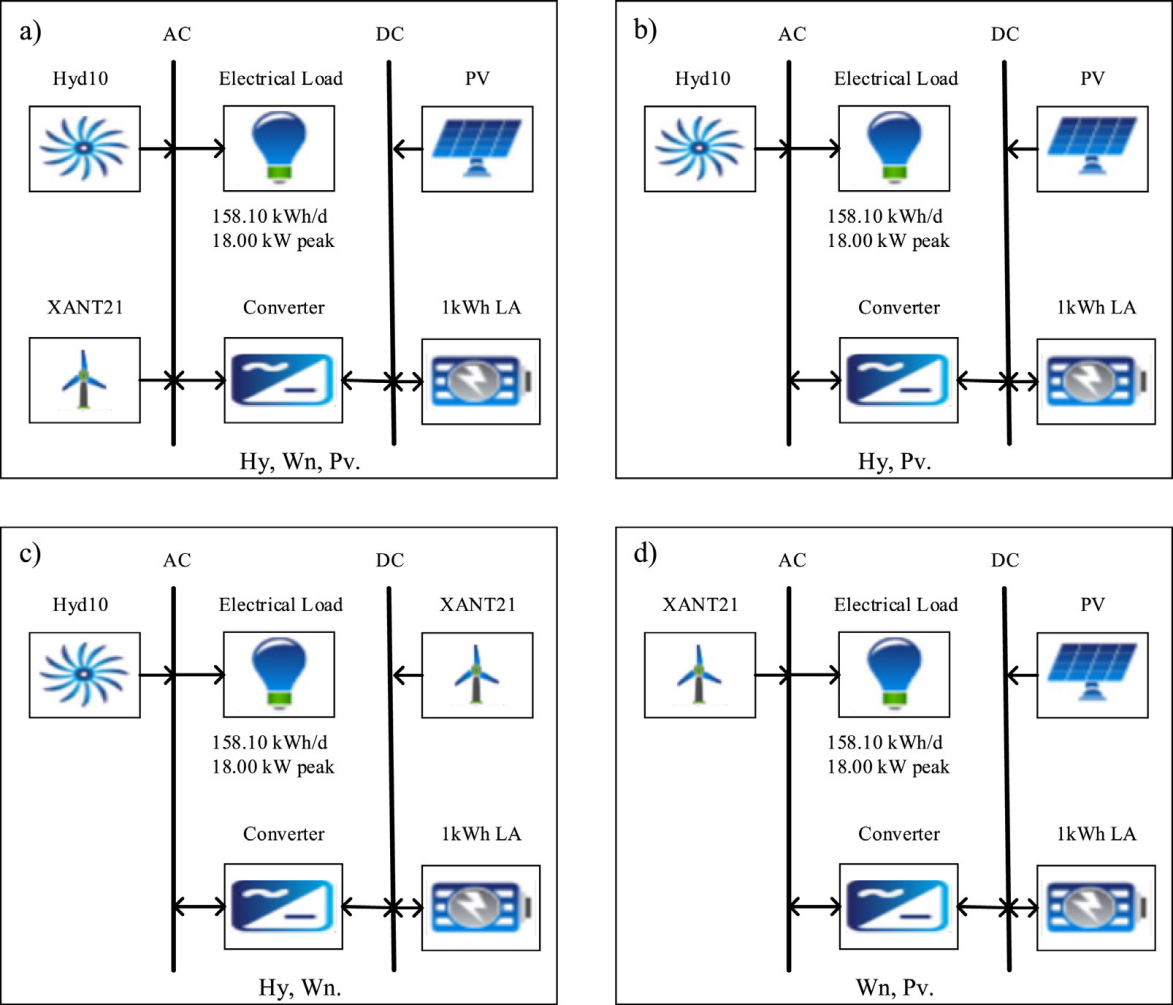
Various configurations of renewable energy hybrid systems: a) is the renewable hydro, wind, and solar photovoltaic hybrid system configuration; b) is the renewable hydro and solar photovoltaic hybrid system configuration; c) is the renewable hydro and wind hybrid system configuration; while the renewable wind and solar photovoltaic hybrid system configuration is shown in d). Abbreviations: Hy: hydro, Wn: wind, Pv: solar photovoltaic.

Any hybrid energy system combines two or more sources of energy, energy storage and power conditioning equipment [61, 62]. In this study, we chose all hybrid system models to contain a micro-hydro combined with other natural resources like wind and solar as shown in Figure 10.

#### 3.7.2 Load details

Using HOMER software, we specify an amount of primary load in kilowatts for each hour of the year, either by importing a file containing hourly data or by allowing HOMER to synthesize hourly data from average daily load profiles. Therefore, HOMER creates hourly load values based on user-specified daily load profiles [45].

In this paper, the hybrid system was designed and performance evaluation was conducted taking into consideration the following assumptions:

(1) The primary load of 158.1 kWh/day, and 18.0 kW peak load were assumed in this simulation. However, during the survey conducted in this research, 158.1kWh was estimated as the daily load consumption of 101 households in the countryside as shown in Table 3.
(2) This project lifetime was estimated based on the guarantee of components which are estimated around 25 years.

**Figure 11:**
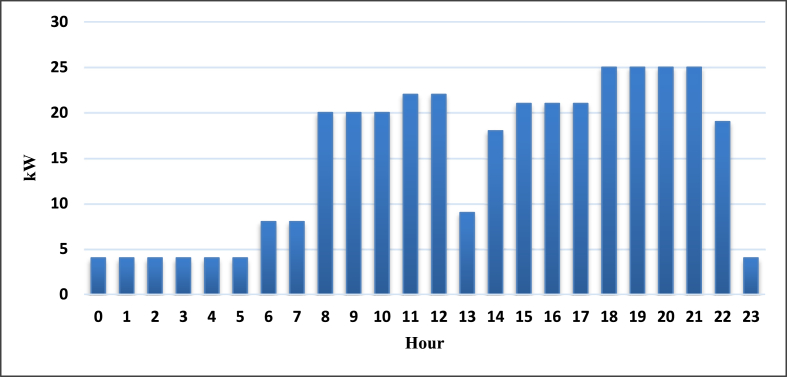
Daily load profile for the selected hybrid systems.

In carrying out the simulations different inputs were required. In this study, the village primary load requirement was carefully estimated considering the data from three off-grid mini-grids visited before the first draft of this paper. We also consulted previously published literature on Rwanda villages. Moreover, the advice and information from solar home systems and mini-grids experts were sought in this research. As a result, many small off-grid villages without high load business customers are electrified using 18 kW (peak) which corresponds roughly to the primary load input of 158.1kWh/day in this simulation. Figure 11 shows the daily load profile.

#### 3.7.3 Natural resources assessment

HOMER uses four RE resources like solar, wind, hydro, and biomass including any fuels used by the components of the system [45, 46]. RE resources are enormous in Rwanda, and this makes rural electrification possible in off-grid villages of Rwanda.

**Figure 12:**
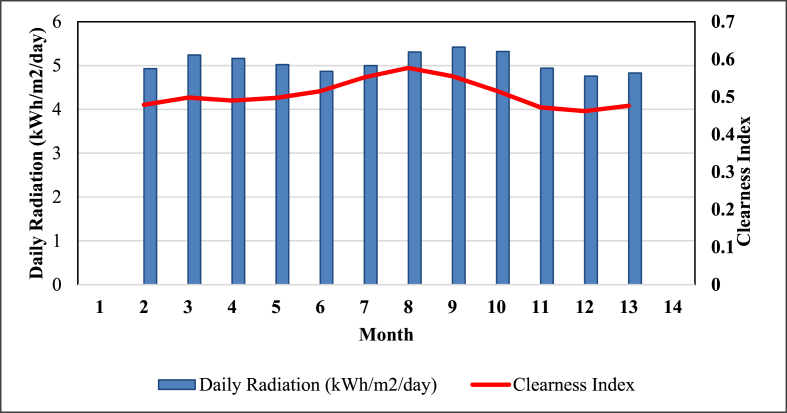
Solar energy profile.

**Figure 13:**
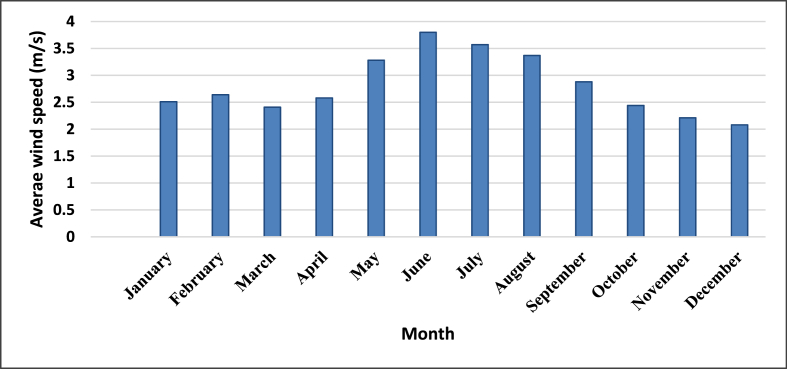
Wind energy profile.

Although we visited 3 off-grid power plants, the hybrid simulation of one power plant location has been used as a test case in this study. Thus, Mukungu pico-hydropower plant resources have been used in this simulation. The daily radiation and average wind speed at the selected village are shown in Figure 12 and Figure 13, respectively. The solar and wind resources data for Mukungu village were obtained from NASA surface meteorology and solar website [63].

The power output of PV array depends on the radiation striking the surface, which is generally not horizontal. HOMER evaluates the output of PV array ${\text{P}}_{\text{PV}}$; and clearness index K_T_ using [45]:

(4) $$P_{PV}=Y_{PV}f_{PV}\left(\frac{{\bar{G}}_{T}}{{\bar{G}}_{T,STC}}\right)\left[1+\alpha_{P}\left(T_{c}-T_{c,STC}\right)\right]$$

If we choose not to model the effects of temperature on the PV array, HOMER assumes the temperature power coefficient is zero. Then, Eq. (4) simplifies to [45]:

(5) $$P_{PV}=Y_{PV}f_{PV}\left(\frac{{\bar{G}}_{T}}{{\bar{G}}_{T,STC}}\right)$$

(6) $$K_{T}=\frac{H_{ave}}{H_{o,ave}}$$

where: ${\text{Y}}_{\text{PV}}$ is rated capacity of PV array (kW); ${\text{f}}_{\text{PV}}$ is PV derating factor (%); ${\bar{\text{G}}}_{\text{T}}$ is incident solar radiation on PV array in current time (kilowatt per square metre) (kW/m^2^); ${\bar{\text{G}}}_{\text{T},\text{STC}}$ is incident radiation at standard test conditions (1 kW/m^2^); $\alpha_{\text{P}}$ is power temperature coefficient (%/°C); ${\text{T}}_{\text{c}}$ is current time step PV cell temperature (degree Celsius) (°C); ${\text{T}}_{\text{c},\text{STC}}$ is standard PV cell temperature test conditions (at 25 °C); ${\text{H}}_{\text{ave}}$ is earth horizontal surface monthly average radiation (kWh/m^2^/day); and ${\text{H}}_{\text{o},\text{ave}}$ is extraterrestrial horizontal radiation (kWh/m^2^/day).

In the case of wind power resource, HOMER determines wind turbine power output in each time step using a three-step process [45]. First, it evaluates wind speed at the hub height of the wind turbine. Second, it assesses the corresponding wind speed at the turbine's hub height using either the logarithmic or power law. Third, it uses the turbine's power curve to determine how much power the wind turbine produces at that wind speed at standard air density conditions. Finally, it modifies the power output value for the authentic air density. Wind speed equations at the hub height of wind turbine ${\text{U}}_{\text{hub}}$ and wind turbine power output ${\text{P}}_{\text{WTG}}$, become [45]:

(7) $$U_{hub}=U_{anem}\;.\;\frac{ln\left(Z_{hub}/Z_{0}\right)}{ln\left(Z_{anem}/Z_{0}\right)}$$

If we apply the power law, HOMER determines ${\text{U}}_{\text{hub}}$ using [48]:

(8) $$U_{hub}=U_{anem}\;.{\left(\frac{Z_{hub}}{Z_{anem}}\right)}^{\alpha }$$

(9) $$P_{WTG}=\left(\frac{\rho }{\rho_{0}}\right).P_{WTG,STP}$$

where: ${\text{U}}_{\text{anem}}$ is wind turbine wind speed at hub height (m/s); ${\text{Z}}_{\text{hub}}$ is hub height of wind turbine (m);${\text{ Z}}_{\text{anem}}$ is anemometer height (m); ${\text{Z}}_{0}$ is surface roughness length (m); ln(..) is natural logarithm; α is power law exponent; ${\text{P}}_{\text{WTG},\text{STP}}$ is standard temperature and pressure wind turbine power output (kW); ρ is actual air density (kilogram per cubic metre) (kg/m^3^); and $\rho_{0}$ is air density at standard temperature and pressure (1.225 kg/m^3^).

**Figure 14:**
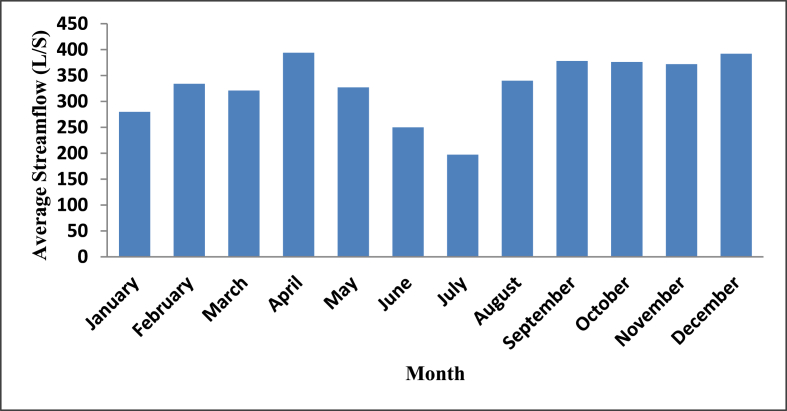
Hydro energy profile at the selected village.

In the case of hydropower resource, the average monthly stream-flow data were obtained. During the site visit and survey of Mukungu power plant, the owners of this project said the data for hydropower resource was obtained between 2014 and 2016 in the course of the feasibility study of this power plant. In each step, HOMER evaluates the electrical power output of the hydro turbine using [45]:

(10) $$P_{hyd}=\frac{\eta_{hyd}\rho_{water}gh_{net}{\dot{Q}}_{turbine}}{1000W/kW}$$

where: $\eta_{\text{hyd}}$ is hydro turbine efficiency (%); $\rho_{\text{water}}$ is density of water (kg/m^3^); g is acceleration due to gravity (9.81 m/s^2^); ${\text{h}}_{\text{net}}$ is effective head (m); and ${\dot{\text{Q}}}_{\text{turbine}}$ is hydro turbine flow rate (m^3^/s). Also, HOMER determines the effective head (or net head) ${\text{h}}_{\text{net}}$ and flow through the turbine ${\dot{\text{Q}}}_{\text{turbine}}$ using [45,46]:

(11) $$\;\;h_{net}=h\left(1-f_{h}\right)$$

(12) $${\dot{\text{Q}}}_{\text{turbine}}=\{\begin{array}{c} 0\;\;\;\;\;\;\;\;if\;{\dot{Q}}_{available}<{\dot{Q}}_{min}\;\;\;\;\;\;\;\;\;\;\;\;\;\;\;\;\;\;\;\;\;\;\;\;\\ {\dot{Q}}_{available}\;if\;{\dot{Q}}_{min}\leq {\dot{Q}}_{available}\leq {\dot{Q}}_{max}\;\;\;\;\;\;\;\;\;\;\;\;\;\;\;\;\;\\ {\dot{Q}}_{max}\;\;\;\;if\;{\dot{Q}}_{available}>{\dot{Q}}_{max\;\;\;\;\;\;\;\;\;\;}\;\;\;\;\;\;\;\;\;\;\;\;\;\;\;\;\; \end{array}$$

where: h is available head (m); ${\text{f}}_{\text{h}}$ is pipe head loss (%); ${\dot{\text{Q}}}_{\text{available}}\;$is available hydro turbine flow rate (m^3^/s); ${\dot{\text{Q}}}_{\text{min}}$ is minimum hydro turbine flow rate (m^3^/s); and ${\dot{\text{Q}}}_{\text{max}}\text{ is }$maximum hydro turbine flow rate (m^3^/s). Further, Eq. (6) shows that HOMER assumes that unless the available stream flow exceeds the turbine's minimum flow rate, the turbine flow rate is zero. Therefore, the turbine is not operating and is not producing power [46]. Table 3 and Figure 14 show the hydro resources at the selected site.

#### 3.7.4 System components

In this study, we used different components according to the HBT that we want to simulate. The components which generate, convert and store energy were included in this simulation. In HOMER analysis, solar photovoltaic (PV), wind turbines, and run-off river hydropower were the intermittent resources. The batteries and converter are for storing and converting the electricity, respectively. The performance and cost of each of the system's components are major considerations for the desired results in the design. The components' technical and cost parameters for this study were based on data collected from power producers', and mini-grid off-grid private companies in Rwanda and assumptions based on the previously published literature. Table 4 clearly shows each component's performance and cost.

**Table 4:** Systems components and their costs.

No	Component	Rated Capacity	Capital cost (USD)	Replacement cost (USD)	O & M cost/year (USD)	Lifetime (Years)
1	Wind turbine (XANT M-21-ETR [100kW]	100 kW	80,000.00	64,000.00	400.00	25
2	Hydro turbine (10kW Generic)	10 kW	20,000.00	15,000.00	1,500.00	30
3	Converter (System converter)	10 kW	30.000.00	25,000.00	1500.00	15
4	24 Batteries (Generic)	1kWh each	2,400.00	2,000.00	120.00	10
4	PV	10 kW	16,000.00	12,000.00	100.00	25

## 4 Simulation, optimization results, and discussion

Modelling and simulation results were processed using the HOMER micro-power optimization model. For each sensitivity case that HOMER solves, it simulates every system in the search space and ranks all the feasible systems according to increasing net present cost. In the optimization process, HOMER simulates a range of equipment options over varying constraints and sensitivities to optimize small power systems. The optimization tables are used to compare these systems. The optimization table lists architecture data about each system, like number of batteries, size of converter, or capacity of PV. It also provides cost data like Levelized Cost of Energy (LCOE), Net Present Cost (NPC), operating cost, initial capital, and renewable fraction. In this study, four hybrid renewable energy systems were designed using HOMER renewable energy software through a large number of hourly simulations. Different values for wind speed, solar radiation, and hydro resources (average stream-flow) were considered in the simulations and analyses. Table 5 shows the simulation and optimization of 4 different off-grid hybrid systems for the case study without considering the sensitivity variables.

**Table 5:** Comparative simulation analysis without considering sensitivity variables.

Resources/HBT	System architecture	Electricity production (kWh/yr), (Fraction)	Cost summary		
			Total NPC (USD)	LCOE (USD/kWh)	Operating cost (USD/Yr)
Hy,Wn,Pv	Hy turbine: 1 turbine	122,716 : 82.6%	126,324.10	0.1715	1,895.49
	Wn turbine: 1turbine	24,203 : 16.3%			
	Pv: 1.09 kW	1,620 : 1.09%			
	Battery: 2 strings	**X**			
	Converter: 0.05 kW	**X**			
	Total yearly production = 148,539 kWh				
Hy,Pv	Hy turbine: 1 turbine	122,716 : 98.7%	41,210.80	0.05601	1,495.49
	Pv: 1.09 kW	1,620 : 1.3%			
	Battery: 2 strings	**X**			
	Converter: 0.05 kW	**X**			
	Total yearly production = 124,336 kWh				
Hy,Wn	Hy turbine: 1 turbine	122,716 : 83.5%	124,175.90	0.1688	1,873.48
	Wn turbine: 1 turbine	24,2013 : 16.5%			
	Battery: 312 strings	**X**			
	Converter: 13.1kW	**X**			
	Total yearly production = 146,919 kWh				
Wn,Pv	Wn turbine: 1 turbine	24,203 : 73.6%	310,014.20	0.4328	6,780.57
	Pv: 45.5 kW	67,425 : 26.4%			
	Battery: 312 strings	**X**			
	Converter: 13.1 kW	**X**			
	Total yearly production = 91,629 kWh				

Abbreviations: Hy: Hydro, Wn: Wind, Pv: solar PV, yr: years.

The simulation considered different HBT options. As shown in Table 5, the minimum LCOE obtained from the results is USD 0.05601/kWh for hydro and PV combinations. This hybrid system comprises 1 hydro turbine, 1.09 kW PV which will generate 124,336 kWh total yearly production and the hydro turbine and PV production ratios of 98.7% and 1.3%, respectively. The total NPC and operating cost for such hybrid system are USD 41,210.80, and USD 1,495.49/year, respectively.

The most expensive combination of hybrid system comprises a wind turbine and PV. It consists of 45.5 kW PV and 1 wind turbine. The total NPC, LCOE, and operating cost for such hybrid system are USD 310,014.20, USD 0.4328/kWh and USD 6,780.57/year, respectively. The total yearly energy production of this hybrid system is 91,629.0 kWh which includes 73.6% and 26.4% wind turbine and photovoltaic PV production ratios, respectively. Moreover, the pollutant emissions of all the possible hybrid system configurations mentioned in Table 5 are completely reduced.

As access to electricity is the engine for development and improvement of welfare, the government of Rwanda is targeting 100% access to electricity for all population by 2024. Rwanda has abundant natural energy resources including hydro, solar, geothermal, methane gas and wind energy to be investigated before any decision. This can attract energy generation projects which can increase electricity access and generation capacity from 224.6 MW to 556 MW by 2024. Also, the technical analysis from hybrid systems simulations and collected data from different mini-grids indicate that remote areas without hydro resources, it is better to use small solar mini-grids or standalone systems because they can provide electricity more quickly and at a much lower cost.

The challenge of air pollution, climate change, and energy-security problems worldwide need extensive and complete game-changers of the world's energy infrastructure to one hundred per cent clean, renewable energy which produces zero emissions. Currently, 4–7 million people prematurely die annually while hundreds of millions of people are taken ill because of air pollution. These cause enormous pain, suffering and economic costs that could be eliminated by implementing zero-emission energy systems [64]. Similarly, steering clear of 1.5 °C global warming demands from over 80% energy conversion infrastructure to zero-emitting energy by 2030 and one hundred per cent by 2050, would help. Also, fossil-fuel depletion has led to a rise in prices, economic, social, and political instabilities in diverse places except workable energy solutions are developed before the crises occur, the world would be heading towards a catastrophe [64].

In this modern era, worries about climate change are affecting more people as global warming becomes more apparent around the world. However, one way of tackling climate anxiety is to electrify all energy sectors (transportation, heating/cooling, industry, agriculture/forestry/fishing) and provide electricity to all using 100% wind, water, and solar (WWS) power. Electric power is produced at the power stations which are located at favourable places, generally quite far from consumers. It includes a network of transmission lines, power generation stations, substations, and other auxiliary equipment. Often, the power transmitted through the power grid may be disrupted. There are many causes of power failures in an electricity network such as bad weather, man-made faults, equipment malfunctions, and utter disregard for power operations rules and regulations. To reinforce the country's capacity and the risk of large-scale power outages by allowing a stable supply of electricity, every country should establish a roadmap to call upon for electrifying different sectors with WWS. Also, different technology needs like smart meters and IUTs are required because they reduce power outages and restoration times.

Accordingly, a small hybrid-electric system that combines renewable resources technologies offers several advantages over any single system. Renewable hybrid systems such as hydro/solar/battery combinations are suitable optimum solutions for rural electrification because they significantly increase the proportion of renewable electricity resources we use at maximum, and reduce diesel consumption. Also, they reduce the cost of production as well as promote green growth.

## 5 Conclusion

A lack of electricity access is slowing down growth and hindering the achievement of development goals. In this study, four different 100% renewable energy hybrid systems were designed and simulated to provide power to a rural community in Rwanda. The simulation results show that the combination of hydro/solar/battery is the best (or optimal) solution to the off-grid system which can be used in remote areas. The hydropower potential sites are useful because of the reduced NPC, LCOE and zero greenhouse gas emissions and lower carbon footprint. The total NPC, LCOE and operating cost for such hybrid energy system are USD 41,210.80, USD 0.05601/kWh, and USD 1,495.49/year, respectively. This option is cheaper than other hybrid system combinations that contain wind resource as we have seen in Table 5. The optimization and simulation processes were conducted using HOMER software. As the country targets to achieve reliable energy access for all, the selected hybrid energy system might be the solution to rural electrification, mitigate climate change impacts, and lower carbon footprint development. From simulation results, it can be concluded that the proposed hybrid system is the optimal solution to the energy crisis in Rwanda because it will make significant contribution to renewable power generation in future. We also found that any hybrid combination of wind technologies could not be recommended due to the higher LCOE, capital cost of system components, and NPC. Finally, the results presented in this study can apply to other countries all over the world where environment, climate, weather and meteorological conditions are comparable. For instance, the neighbouring countries of Burundi, Uganda, Tanzania, and the Democratic Republic of Congo are prime candidates. Besides, Rwanda power sector is among the fastest-growing; as it provides opportunities to private partners who are interested in electricity needs to be delivered through off-grid solutions such as standalone solar systems and mini-grids (solar, hydro or hybrid). However, the key technological development needs which are discussed in this paper are needed in the country's power sector to confront the several challenges as many people still do not have access to electricity and the incessant power outages for people who have access.

## Declarations

### Author contribution statement

Jean De Dieu Niyonteze, Godwin Norense Osarumwense Asemota, Samuel Bimenyimana & Gilbert Shyirambere: Conceived and designed the experiments; Performed the experiments; Analyzed and interpreted the data; Contributed reagents, materials, analysis tools or data; Wrote the paper.

Fumin Zou: Conceived and designed the experiments; Wrote the paper.

### Funding statement

This work was supported by the Fujian Provincial Department of Science and Technology (No. 2015J01652).

### Competing interest statement

The authors declare no conflict of interest.

### Additional information

No additional information is available for this paper.
